# Chronic Stress Induces Hepatic Steatosis via Brain-Hepatic Sympathetic Axis Mediated Catecholamine Resistance

**DOI:** 10.7150/ijbs.126058

**Published:** 2026-01-08

**Authors:** Shanshan Wu, Jiachen Liu, Shanshan Huang, Yuxin Guo, Yan Bi

**Affiliations:** 1 Department of Endocrinology, Endocrine and Metabolic Disease Medical Center, Nanjing Drum Tower Hospital, Affiliated Hospital of Medical School, Nanjing University, Nanjing, 210008, China.; 2 Branch of National Clinical Research Centre for Metabolic Diseases, Nanjing, 210008, China.

**Keywords:** chronic stress, MASLD, sympathetic nerves, PVH, catecholamine resistance

## Abstract

Chronic stress is epidemiologically linked to metabolic dysfunction-associated steatotic liver disease (MASLD), yet the underlying mechanisms remain unclear. In mice exposed to chronic restraint stress (CRS), we observed weight-independent hepatic steatosis with marked degeneration of sympathetic fibers. Stress elevated circulating norepinephrine levels but blunted hepatic β-adrenergic/cyclic adenosine monophosphate (cAMP) signaling accompanied by downregulation of β3-adrenergic receptor (β3-AR), indicating hepatic catecholamine resistance. Blocking hepatic sympathetic input prevented stress-aggravated steatosis and restored β-adrenergic signaling, whereas pharmacologic activation of β3-AR with mirabegron alleviated stress-induced lipid accumulation. Pseudorabies virus retrograde tracing and neuronal circuit interrogation further showed that projection from the medial central amygdaloid nucleus (CeM) to paraventricular hypothalamic corticotropin-releasing hormone (CRH^PVH^) neurons mediated stress induced hepatic steatosis. Together, these results reveal a CeM-CRH^PVH^-hepatic sympathetic axis that couples central stress signaling to peripheral β-adrenergic desensitization and lipid dysregulation, thereby suggesting a potential therapeutic strategy for stress-related MASLD.

## Introduction

Stress is a common physiological and psychological stimulus that pervades modern life. Chronic or excessive stress disrupts neuroendocrine homeostasis and predisposes individuals to metabolic dysregulation^1^, ^2^. Epidemiological studies report that approximately 50% of patients with metabolic dysfunction-associated steatotic liver disease (MASLD) experience chronic stress; these patients present also exhibit more severe hepatic lipid accumulation, suggesting a close association between stress and MASLD^3^. Obesity and type 2 diabetes, both common comorbidities of MASLD, are likewise associated with chronic stress^4^, ^5^. However, whether stress promotes lipid deposition in liver and the underlying mechanism remains unclear.

Stress activates both the hypothalamic-pituitary-adrenal axis and the autonomic nervous system, thereby modulating metabolic functions of multiple peripheral organs^6^, ^7^. Serving as a major conduit between the central nervous system and peripheral organs, the autonomic nervous system rapidly conveys integrated signals^6^, ^8^. Sympathetic nerves are the predominant autonomic input to the hepatic parenchyma, and the vagal innervation are minimal in the liver parenchyma^9^, ^10^. In adipose tissue, enhanced sympathetic activity has been well established as a key driver of lipolysis, free fatty acid (FFA) release, and the “browning” of white adipose tissue, underscoring the importance of sympathetic networks in lipid metabolism^11^, ^12^. Nevertheless, the structural and functional adaptations of hepatic sympathetic nerves in response to stress, as well as their mechanistic roles in hepatic lipid metabolism, remain largely unexplored.

Catecholamines, such as norepinephrine (NE) and epinephrine, are key mediators of sympathetic control of peripheral energy metabolism^13^. Catecholamine signaling through adrenergic receptors (AR), particularly β3-AR in adipose tissue, promotes lipolysis and energy mobilization^14^-^16^. Emerging evidence indicates that in conditions such as obesity, insulin resistance, and chronic inflammation, peripheral tissues exhibit markedly diminished responsiveness to NE-a phenomenon termed “catecholamine resistance”, i.e., β3-AR desensitization^16^, ^17^. In adipose tissue, catecholamine resistance manifests as impaired lipolysis, limiting lipid mobilization and promoting fat accumulation^16^. Beyond being a characteristic feature of metabolic syndrome, catecholamine resistance may represent a key mechanistic link between neural input and metabolic dysfunction. However, its role in stress-associated hepatic lipid dysregulation remains largely unexplored.

The paraventricular nucleus (PVH) is a critical hub for controlling the autonomic nervous system, regulating brown adipose thermogenesis and white adipose lipolysis via projections to sympathetic preganglionic neurons in the spinal cord^18^, ^19^. It remains to be defined whether the PVH-sympathetic axis contributes to stress-induced hepatic lipid dysregulation and, if so, what mechanisms underlie this effect. Notably, the PVH comprises diverse neuronal subpopulations, including corticotropin releasing hormone (CRH) neurons, arginine vasopressin (AVP) neurons, and oxytocin (OXT) neurons. These neuronal subsets regulate energy expenditure and adipose tissue metabolism either independently or via discrete neural circuits^20^-^22^. Dissecting how specific PVH neuronal populations integrate stress signals and modulate hepatic lipid metabolism though sympathetic output will be key to elucidating the mechanistic link between stress and hepatic lipid homeostasis.

Here, we report that chronic stress induces hepatic steatosis via a medial central amygdaloid nucleus (CeM)-CRH^PVH^-hepatic sympathetic pathway. We further demonstrate that sustained central stress signaling triggers hepatic catecholamine resistance, characterized by β3-adrenergic desensitization and sympathetic structural degeneration. By extending the concept of catecholamine resistance from adipose tissue to the liver, our study identifies a CeM-CRH^PVH^-hepatic sympathetic-catecholamine axis as a mechanistic bridge linking chronic stress to hepatic lipid accumulation.

## Materials and Methods

### Mice

All the mouse experimental procedures were performed in accordance with the Laboratory Animal Care Guidelines approved by the Model Animal Research Center of Nanjing University. 8-week-old male C57BL/6J mice were purchased from Charles River Laboratory (Beijing, China). Mice were kept under standard conditions with temperature (22 ±1°C) and humidity (~40%) in a 12-hour light/12-hour dark cycle, with food and water provided ad libitum.

### Chronic restraint stress (CRS)

CRS in rodents is widely used to model the negative impact of chronic psychological stress^23^, ^24^. Mice were fed with normal diet (ND) or high-fat diet (HFD, 60% fat) and subjected to CRS stress by placement in 50-ml conical tubes with holes for air flow for 2-4 h per day for 14 consecutive days.

### Behavioral analysis

Open-field test (OFT) and force-swim test (FST) were used to model behavioral despair as previously described^23^, ^25^. All behavioral testing began by allowing the mice to habituate in the test rooms for 2 h before tests. Video data were analyzed by Panlab SMART v3.0.

For OFT, mice were placed in the center of the arena (40 cm x 40 cm x 40 cm) and allowed to explore freely for 5 minutes. Distance traveled and the duration of time spent in the center (20 cm x 20 cm) were analyzed. The boxes were cleaned with 75% ethanol between different mice.

For FST, mice were individually placed in a cylinder (12 cm diameter, 25 cm height) of water (23-25 °C) and swam for 6 minutes. The behaviors were videotaped from the side. The duration of immobility was defined as the time when animals remained floating or motionless with only movements necessary for keeping balance in the water. The duration of immobility during minutes 2-6 was recorded.

### Body weight and food intake

Body weights of mice were recorded at 2-day intervals during CRS. Daily food intake was calculated by cages and then divided by the number of mice per cage to calculate daily food intake per mouse.

### Sympathetic nerve blockade

6-hydroxydopamine hydrobromide (6-OHDA, 162957, Sigma, Germany) solution was prepared freshly by dissolving 6-OHDA in 0.1% ascorbic acid in 0.9% sterile NaCl. For ablation of hepatic sympathetic nerves, 6-OHDA (100 mg/kg body weight) was intraperitoneal injected 30 minutes before CRS on the first 3 days of each week. Control mice were injected with an equivalent volume of vehicle (0.1% ascorbic acid in 0.9% sterile NaCl). For sympathetic ablation of adipose tissue, 8ul of 6-OHDA (12mg/ml) was injected into subcutaneous adipose tissue (sWAT) and epididymal adipose tissue (eWAT) of each mouse. Control mice were injected with an equivalent volume of vehicle. Follow-up experiments were performed two weeks after surgery.

### β3-AR activation

For active β3-AR, mirabegron (SML2480, Sigma, Germany) solution was prepared freshly by dissolving mirabegron in 5% dimethyl sulfoxide (DMSO), During CRS, mirabegron (0.8 mg/kg body weight) was administered to mice by gavage 30 minutes before CRS each day. Control mice were administered with an equivalent volume of vehicle (5% DMSO).

### Virus information and stereotaxic surgery

All virus were generated and purified by BrianVTA (Wuhan, China). For retrograde tracing of hepatic nerves, a total of 1E+10^9^ vg (5ul) pseudorabies virus (PRV)-CAG-EGFP were injected into the left lobe of liver for 5 injecting sites (1 μl per point) over 30 seconds using a Hamilton syringe, and the needle was left in place for 1 additional minute. To inhibit the activity of PVH neurons, a total of 0.3 μl (1E+12v.g/ml) rAAV-hSyn-hM4D(Gi)-mCherry-WPRE polyA (rAAV-hSyn-EYFP-WPRE-hGH polyA as control) were stereotaxic injected into the PVH region, mice in each group were intraperitoneally injected with clozapine-N-oxide (CNO) 30 minutes before daily CRS. To inhibit the activity of CeM-PVH projection, 0.3 μl (1E+12v.g/ml) of rAAV-hSyn-DIO-hM4D(Gi)-mCherry-WPRE polyA (rAAV-hSyn-DIO-mCherry-WPRE polyA as control) were injected in to CeM, and Retro-Cre-WPRE-polyA were stereotaxic injected into the PVH, mice in each group were intraperitoneally injected with CNO 30 minutes before daily CRS. To inhibit the activity of CRH neurons in PVH, 0.3 μl (1E+12v.g/ml) virus mixture of DIO-hM4D(Gi)-mCherry-WPRE polyA (rAAV-hSyn-DIO-mCherry-WPRE polyA as control) and rAAV-hSyn-CRH Cre WPRE polyA were injected into the PVH, mice in each group were intraperitoneally injected with CNO 30 minutes before daily CRS. Injected mice were perfused at specified time points, only animals with the correct injection site were included in the study.

### Liver optical clearing and immunolabeling

Mice were anesthetized and perfused transcardially with PBS (10 U/mL heparin) followed by 4% paraformaldehyde (PFA). Livers were post-fixed in 4% PFA overnight at 4°C and washed thoroughly in PBS. Tissues were delipidated in clearing solution A (NH-CR-210701, Nuohai Life Science, Shanghai, China) at 37°C with daily solution changes for 7 days, then washed extensively in PBS. For immunolabeling, samples were blocked and incubated with tyrosine hydroxylase (TH, AB152, Millipore, USA) antibodies (4-14 days, 4°C) followed by secondary antibodies (4-7 days, 4°C) in blocking buffer (PBS, 0.1% Triton X-100, 2% BSA, 0.05% sodium azide). After labeling, tissues were washed in PBS and stored protected from light until imaging.

### 3D lightsheet imaging

Immunolabeled tissues were incubated in clearing solution B (NH-CR-210701, Nuohai Life Science, Shanghai, China) at 25°C until optically transparent. Samples were embedded in 2% agarose prepared in clearing solution B and mounted on the LS18 light-sheet microscope. Imaging was performed in the manufacturer's imaging medium under 4× magnification, using global scanning to acquire volumetric datasets. Image reconstruction and 3D visualization were conducted with the manufacturer's software and further analyzed in AMIRA (Visage imaging, Australia).

### RNA extraction and quantitative RT-PCR assay

Total RNA was isolated from liver using TRIzol reagent (15596018CN, Invitrogen) according to the manufacturer's instructions. RNA concentration was determined by NanoDrop Microvolume (Thermo Fisher Scientific). For quantification, 5μg total RNA was used to synthesize cDNA using a reverse transcript regent kit (11142ES60, Yeasen, Shanghai). The quantitative RT-PCR was performed using an SYBR Green mix (11201ES08, Yeasen, Shanghai) on a Light Cycler 480 system (Roche Switzerland).

Primers used in quantitative RT-PCR assay as follow: Glyceraldehyde-3-phosphate dehydrogenase (GAPDH, Forward: AGGTCGGTGTGAACGGATTTG, Reverse: TGTAGACCATGTAGTTGAGGTCA); 3-droxyacyl-CoA dehydrogenase 4 (Hacd4, Forward: AGCCCAGGTATAGGAAGAATGT, Reverse: CCGCATAACTAACCCAATAGCG); Stearic acyl-CoA desaturase 1 (Scd1, Forward: AAGATATTCACGACCCCACC, Reverse: CAGCCGTGCCTTGTAAGTTC); Stearic acyl-CoA desaturase 2 (Scd2, Forward: GCATTTGGGAGCCTTGTACG, Reverse: AGCCGTGCCTTGTATGTTCTG); Phosphatidate phosphatase 1 (Lpin1, Forward: CATGCTTCGGAAAGTCCTTCA, Reverse: GGTTATTCTTTGGCGTCAACCT); Fatty Acid Desaturase 1(Fads1, Forward: AGCACATGCCATACAACCATC, Reverse: TTTCCGCTGAACCACAAAATAGA); Lipase (Lipe, Forward: CCAGCCTGAGGGCTTACTG, Reverse: CTCCATTGACTGTGACATCTCG); Patatin-like phospholipase domain-containing protein 2 (Pnpla2, Forward: GGATGAAAGAGCAGACGGGTAG, Reverse: CGCAAGACAGTGGCACAGAG).

### Immunofluorescence staining

Mice were anesthetized and brains were harvested after perfusion with phosphate-buffered saline (PBS) and 4% paraformaldehyde sequentially. For brain sections, brains were maintained in 4% paraformaldehyde overnight at 4 °C and then soaking in 20% and 30% sucrose solution for dehydration. 20 μm thickness brain sections were collected using a Leica cryostat (Leica CM1950) and incubated in 5% bovine serum albumin with 0.3% Triton-X 100 in PBS for 1 h before incubation with anti-adrenocorticotropic hormone releasing factor (CRF, the precursor of CRH, 1:100, ab272391, Abcam, UK), anti-AVP (1:500, ab213708, Abcam, UK) or anti-OXT (1:500, ab212193, Abcam, UK) antibodies at 4 °C overnight. After 3 washes, the sections were incubated with appropriate fluorescent secondary antibodies for 1 h at room temperature. Sections was stained with 4',6-diamidino-2-phenylindole (DAPI, G1012, Servicebio, Wuhan, China) before panoramic scan using Olympus Slide Scanner (Slideview, VS200, Tokyo).

### Histological procedures

For Hematoxylin & Eosin (HE) staining, liver samples were fixed in 4% paraformaldehyde overnight (at least 24h), followed by dehydration with 70%, 80% and 90% alcohol, and finally embedded in paraffin. 5 μm liver sections were stained with hematoxylin and eosin alcoholic.

For Oil Red O staining, frozen liver samples were cut in sections of 8 μm and stained in filtered Oil red O working solution for 10 minutes. Rinsed sections in distilled water, then counterstained with hematoxylin for 5 minutes and finally mounted in aqueous mounting (glycerin jelly).

For immunohistochemistry staining, brain sections were incubated with c-Fos antibody (1:800, ab208942, Abcam, UK), liver sections were incubated with TH (1:300, AB152, Millipore, USA). Sections incubated with antibodies were visualized with the 3,3'-diaminobenzidine (DAB) chromogen kit (ZLI-9018, ZSGB-BIO). Images were acquired by an Olympus slide scanner (Slideview, VS200, Tokyo).

### Biochemical measurements

Serum alanine aminotransferase (ALT), aspartate aminotransferase (AST), triglycerides (TG) and cholesterol (TC) levels were determined using an automatic biochemical analyzer according to the manufacturer's intructions (Chemray 800, Rayto, Shenzhen, China). Liver TG were measured using triglycerides kit (S03027, Rayto, Shenzhen, China) according to the manufacturer's intructions. Serum leptin (E-EL-M3008), adiponectin (E-EL-M0002), resistin (E-EL-M3056), norepinephrine (NE, E-EL-0047), and hepatic cyclic adenosine monophosphate (cAMP, E-EL-0056) in liver extracts were measured using enzyme-linked immunosorbent assay (ELISA) kits according to the manufacturer's intructions (Elabscience, Wuhan, China). Free fatty acid (FFA, E-BC-K792-M) in serum was measured using the colorimetric method according to the the manufacturer's intructions (Elabscience, Wuhan, China).

### Statistical analysis

Statistical analysis and graphical presentations were generated using SPSS (Version 26.0, IBM SPASS, Chicago, USA), GraphPad Prism (Version 9.4.0, GraphPad Software, San Diego, USA), Adobe Illustrator 2022 (Version 26.3.1, Adobe, San Jose, USA), and Adobe Photoshop 2022 (Version 23.4.1, Adobe, San Jose, USA). Data are presented as the mean ± SEM. Normality tests were applied prior to statistical analysis. Unpaired two-tailed Student's *t*-tests and one-way analysis of variance (ANOVA) were used for statistical analysis. Sample size and detailed statistical information are provided in the figure legends. Differences were considered statistically significant at *P <0.05.

## Results

### Chronic stress exacerbates hepatic steatosis in ND-fed mice

**Figure 1:**
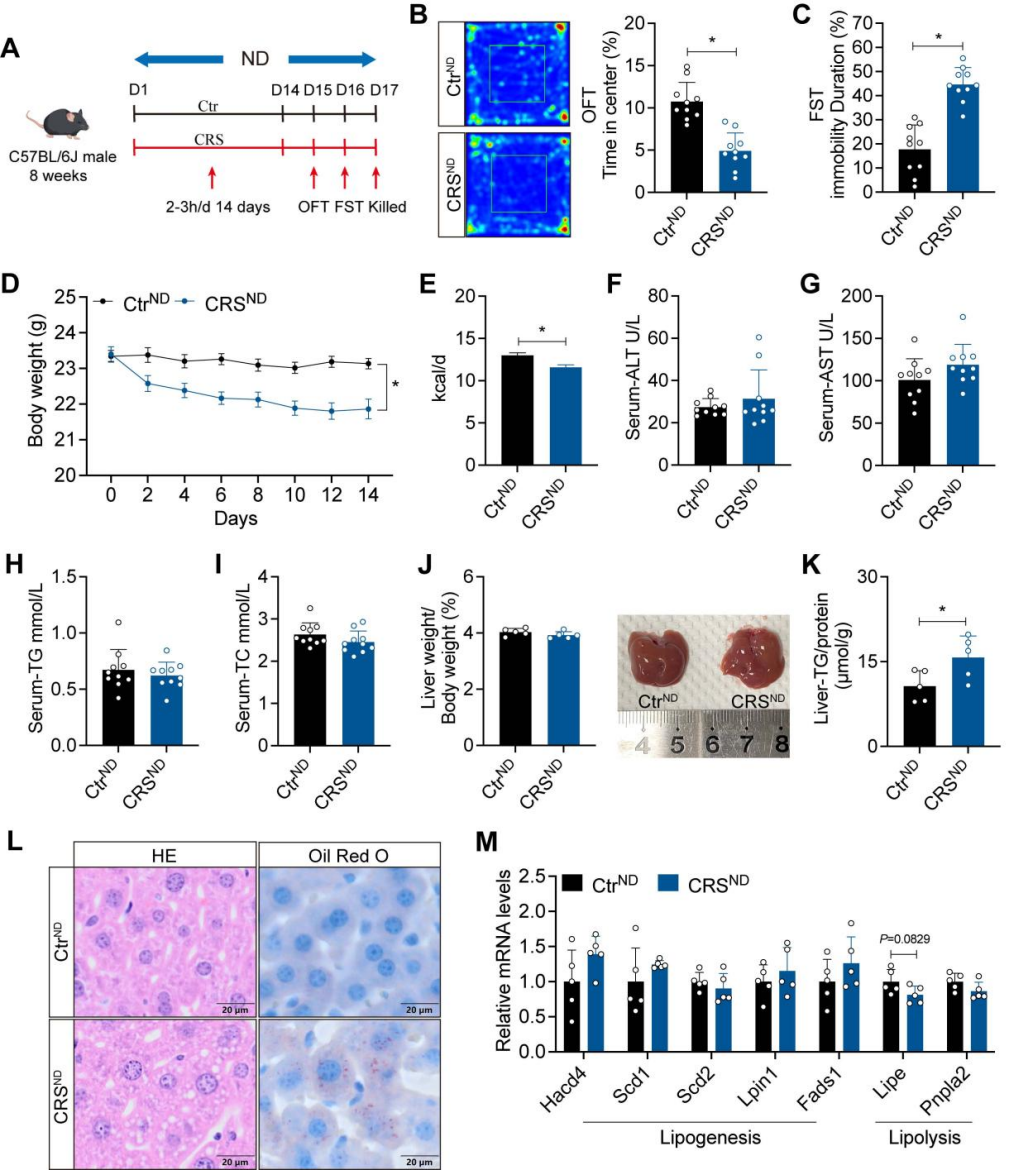
** Chronic stress exacerbates hepatic steatosis in ND-fed mice.** (A) Experimental scheme of CRS mice. (B) Time in center (%) in OFT, n = 10 per group. (C) Immobility duration in FST, n = 10 per group. (D) Body weight, n = 10 per group. (E) Food intake, n = 10 per group. (F-I) Serum ALT, AST, TG and TC concentrations, n = 10 per group. (J) Liver weight and liver anatomy, n = 5 per group. (K) Liver TG, n = 5 per group. (L) H&E staining (scale bars, 20 μm) and Oil Red O staining (scale bars, 20 μm) of liver sections, n = 5 per group. (M) Relative mRNA levels in liver, n = 5 per group. The data are presented as mean ± SEM. *P < 0.05. Unpaired two-tailed Student's *t*-test and one-way ANOVA were used for statistical analysis.

To investigate the impact of chronic stress on MASLD, we subjected ND-fed mice to CRS for 14 days (Figure 1A). CRS mice exhibited pronounced depression-like behaviors, indicating successful establishment of the chronic stress model (Figure 1B, C). Consistent with previous findings^26^, CRS mice showed reduced food intake and body weight (Figure 1D, E) accompanied by unaltered ALT, AST, TG, and TC levels in serum (Figure 1F-I). Although liver weight did not differ significantly between groups, livers from CRS mice were lighter (Figure 1J). Consistently, CRS mice exhibited increased hepatic TG levels and more severe hepatic steatosis (Figure 1K, L). Although changes in gene expression did not reach statistical significance, we observed trends toward increased expression of lipogenesis-related genes and decreased expression of lipolysis-related genes (Figure 1M).

### Chronic stress impairs hepatic lipid homeostasis in HFD-fed mice

**Figure 2:**
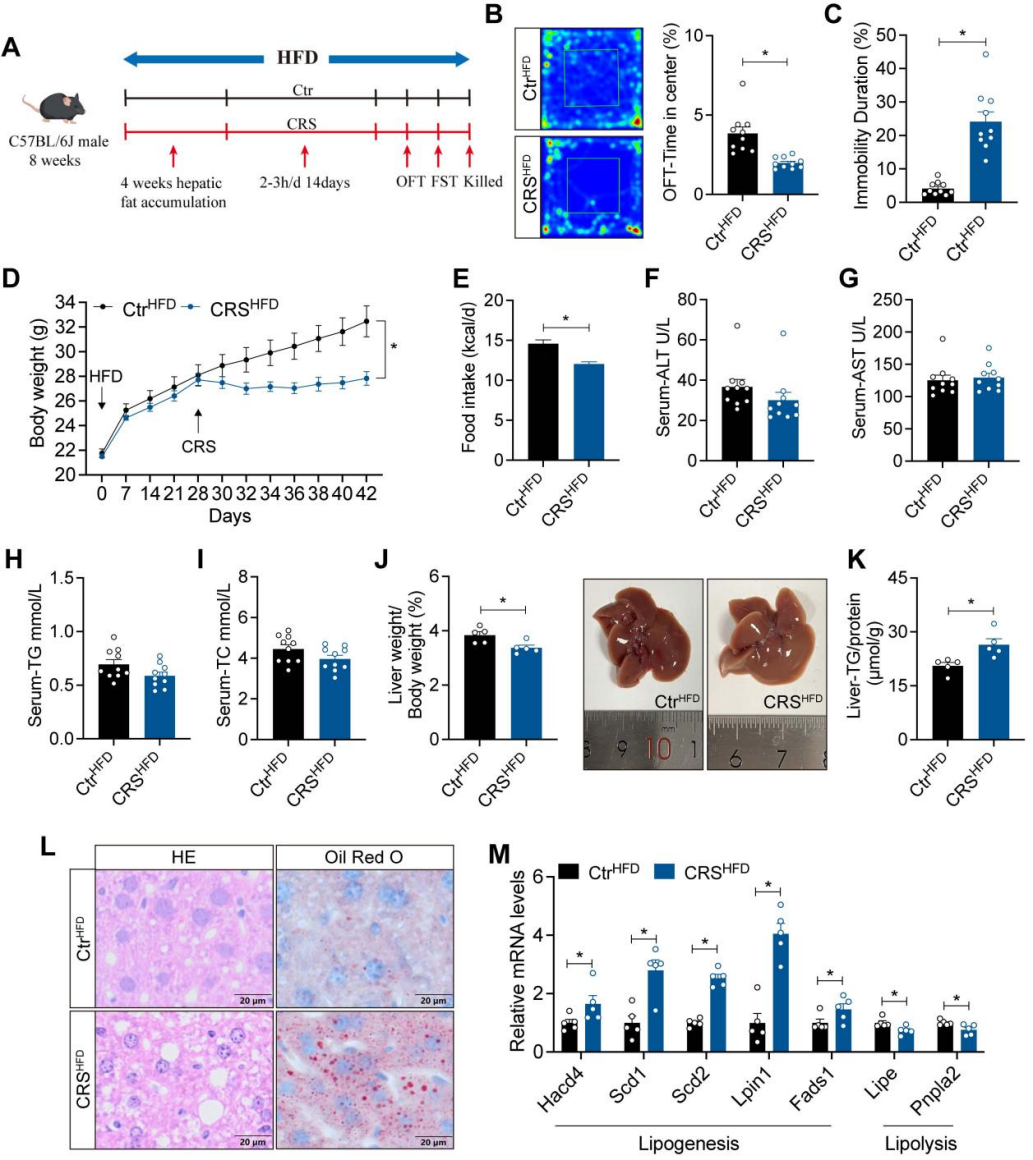
** Chronic stress impairs hepatic lipid homeostasis in HFD-fed mice.** (A) Experimental scheme of CRS mice. (B) Time in center (%) in OFT, n = 10 per group. (C) Immobility duration in FST, n = 10 per group. (D) Body weight, n = 10 per group. (E) Food intake, n = 10 per group. (F-I) Serum ALT, AST, TG and TC concentrations, n = 10 per group. (J) Liver weight and liver anatomy, n = 5 per group. (K) Liver TG, n = 5 per group. (L) H&E staining (scale bars, 20 μm) and Oil Red O staining (scale bars, 20 μm) of liver sections, n = 5 per group. (M) relative mRNA levels in liver, n = 5 per group. The data are presented as mean ± SEM. *P < 0.05. Unpaired two-tailed Student's *t*-test and one-way ANOVA were used for statistical analysis.

To enhance the effects of chronic stress on hepatic lipid metabolism, we fed mice with HFD for 4 weeks before CRS (Figure 2A). Similarly, CRS mice exhibited depressive-like behaviors (Figure 2B, C) and showed significant reductions in body weight and food intake (Figure 2D, E), while serum biochemical parameters remained unchanged (Figure 2F-I). Notably, CRS mice displayed more pronounced hepatic lipid deposition (Figure 2J-L), accompanied by increased expression of lipogenesis-related genes and decreased expression of lipolysis-related genes (Figure 2M). Collectively, these results indicate that chronic stress accelerates MASLD progression in a manner independent of changes in body weight.

### Blockade of adipose sympathetic nerve does not prevent chronic stress-induced impairment of hepatic lipid homeostasis

**Figure 3:**
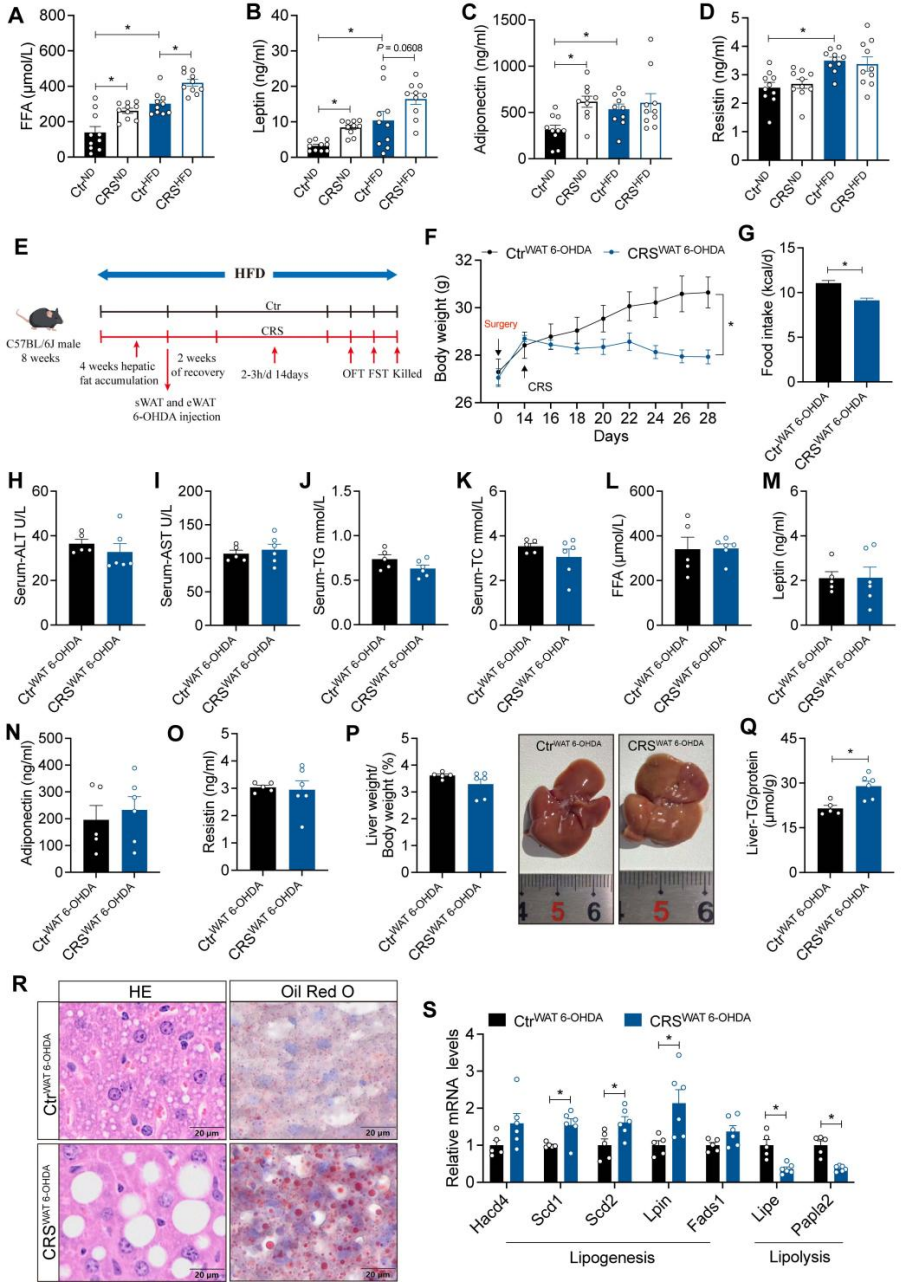
** Blockade of adipose sympathetic nerve does not prevent chronic stress-induced impairment of hepatic lipid homeostasis.** (A-D) FFA, leptin, adiponectin, resistin levels in serum, n = 10 per group. (E) Experimental scheme. (F) Body weight, n= 5:6. (G) Food intake, n = 5:6. (H-K) Serum ALT, AST, TG and TC concentrations, n = 5:6. (L-O) FFA, leptin, adiponectin, resistin levels in serum, n = 5:6. (P) Liver weight and liver anatomy, n = 5:6. (Q) Liver TG, n = 5:6. (R) H&E staining (scale bars, 20 μm) and Oil Red O staining (scale bars, 20 μm) of liver sections, n = 5:6. (S) Relative mRNA levels in liver, n = 5:6. The data are presented as mean ± SEM. *P < 0.05. Unpaired two-tailed Student's *t*-test and one-way ANOVA were used for statistical analysis.

To address whether chronic stress-induced hepatic steatosis is driven by adipose sympathetic activation and subsequent lipid mobilization, we first profiled circulating lipid-related parameters under ND and HFD conditions. CRS significantly altered systemic lipid homeostasis, as evidenced by increased plasma free fatty acids (FFA) (Figure 3A) and concomitant dysregulation of adipokines, including changes in leptin, adiponectin, and resistin (Figure 3B-D), indicating that CRS robustly disrupts peripheral lipid metabolic signals.

Next, we evaluated the causal contribution of white adipose tissue sympathetic activity by chemically denervating sWAT and eWAT (Figure 3E). The results showed that CRS still elicited marked reductions in body weight and food intake (Figure 3F-G) without significant changes in circulating parameters (Figure 3H-K). Importantly, circulating FFA leves and adipokines were not significantly different between the two groups after sympathetic denervation (Figure 3L-O). Nevertheless, CRS continued to promote hepatic lipid accumulation (Figure 3P-S). Thus, suppression of adipose sympathetic input was insufficient to prevent stress-related hepatic steatosis.

### Chronic stress induces severe degeneration of hepatic sympathetic nerves

**Figure 4:**
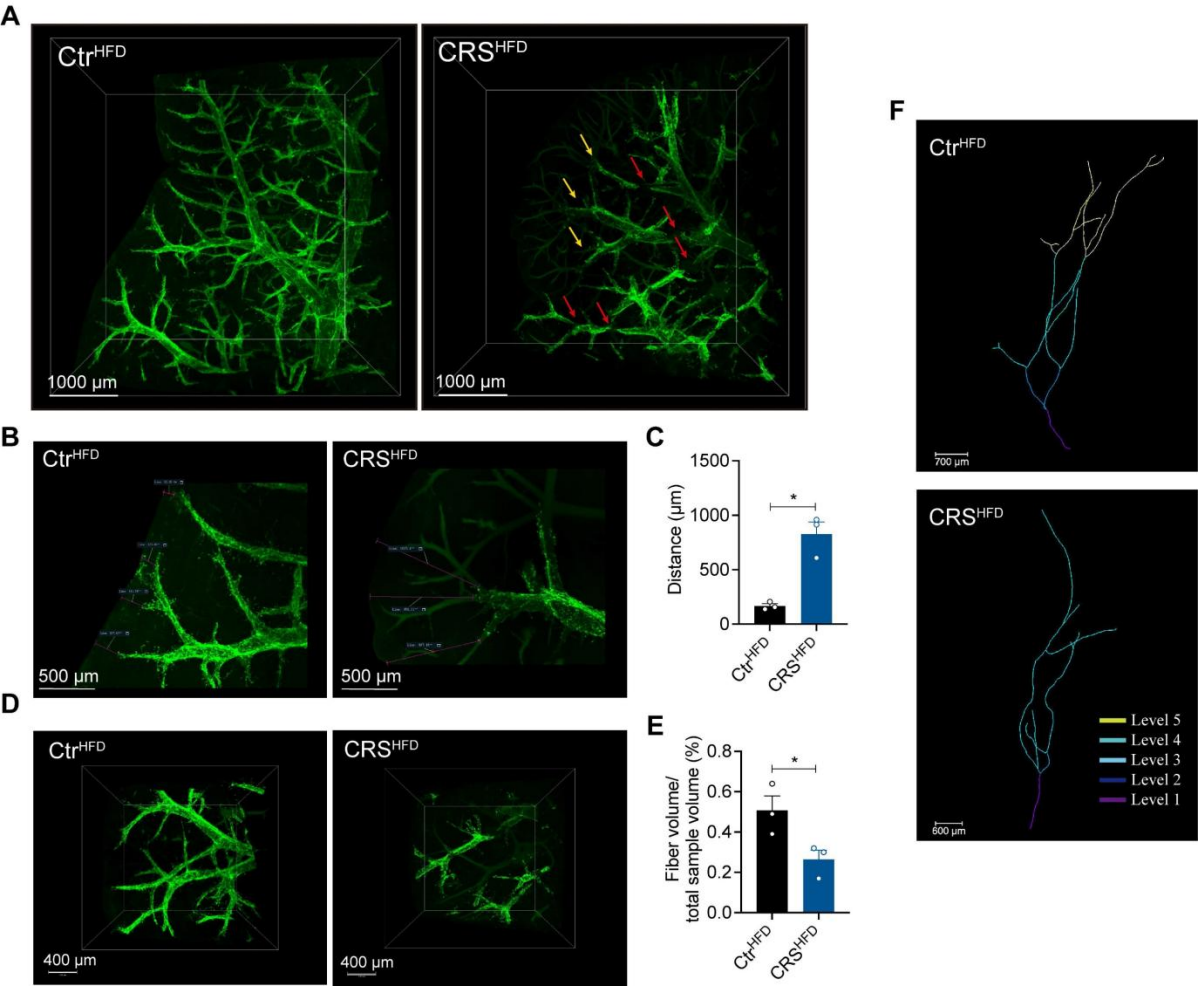
** Chronic stress induces severe degeneration of hepatic sympathetic nerves.** (A) Hepatic sympathetic nerves 3D staining (TH, green, scale bars, 1000 μm), terminal contraction (yellow arrows), fiber breakage (red arrows), n = 3 per group. (B, C) Quantification of TH^+^ fiber ending corresponding to liver surface distances (scale bars, 500 μm), locally enlarged from panel A. (D) Sympathetic nerve fibers of the same volume in the liver (scale bars, 400 μm). (E) Quantification of total sympathetic fiber volume in in the liver of CRS and control mice. (F) Representative examples for traced main fiber arborizations in entire samples (scale bars, 700 μm and 600 μm). The data are presented as mean ± SEM. *P < 0.05. Unpaired two-tailed Student's *t*-test was used for statistical analysis.

Given that sympathetic nerves are key regulators of hepatic metabolism, we next examined whether chronic stress alters hepatic sympathetic innervation^9^, ^10^. We used whole-mount immunostaining and tissue clearing combined with light-sheet fluorescence microscopy to characterize the three-dimensional organization of hepatic innervation in CRS mice. This approach enabled whole-tissue immunolabeling and optical clearing of intact, unsectioned liver samples^9^, ^10^. Using TH immunolabeling, we observed that sympathetic nerves in the mouse liver were predominantly distributed around blood vessels. Compared with the controls, hepatic sympathetic nerve in CRS mice exhibited pronounced structural degeneration, including terminal retraction and fiber fragmentation (Figure 4A).

Quantitative analysis revealed that the average distance between TH positive fiber endings and the liver surface was significantly increased in the CRS group, indicating that CRS caused retraction of hepatic sympathetic nerve terminals (Figure 4B, C). Within an equivalent tissue volume, CRS mice showed a notable decrease in TH positive fiber volume (Figure 4D, E). In addition, marked fiber fragmentation of the hepatic sympathetic nerves was evident in CRS mice (Figure 4D, right). Plotting the branching angles revealed that, in particular, branches with higher branching angles decreased in number, indicating a preferential loss of distal fiber branches (Figure 4F). Together, these findings indicate that chronic stress leads to severe degeneration of hepatic sympathetic nerves, which may contribute to stress-related hepatic lipid accumulation.

### Sympathetic hyperactivity mediates stress-induced hepatic steatosis

**Figure 5:**
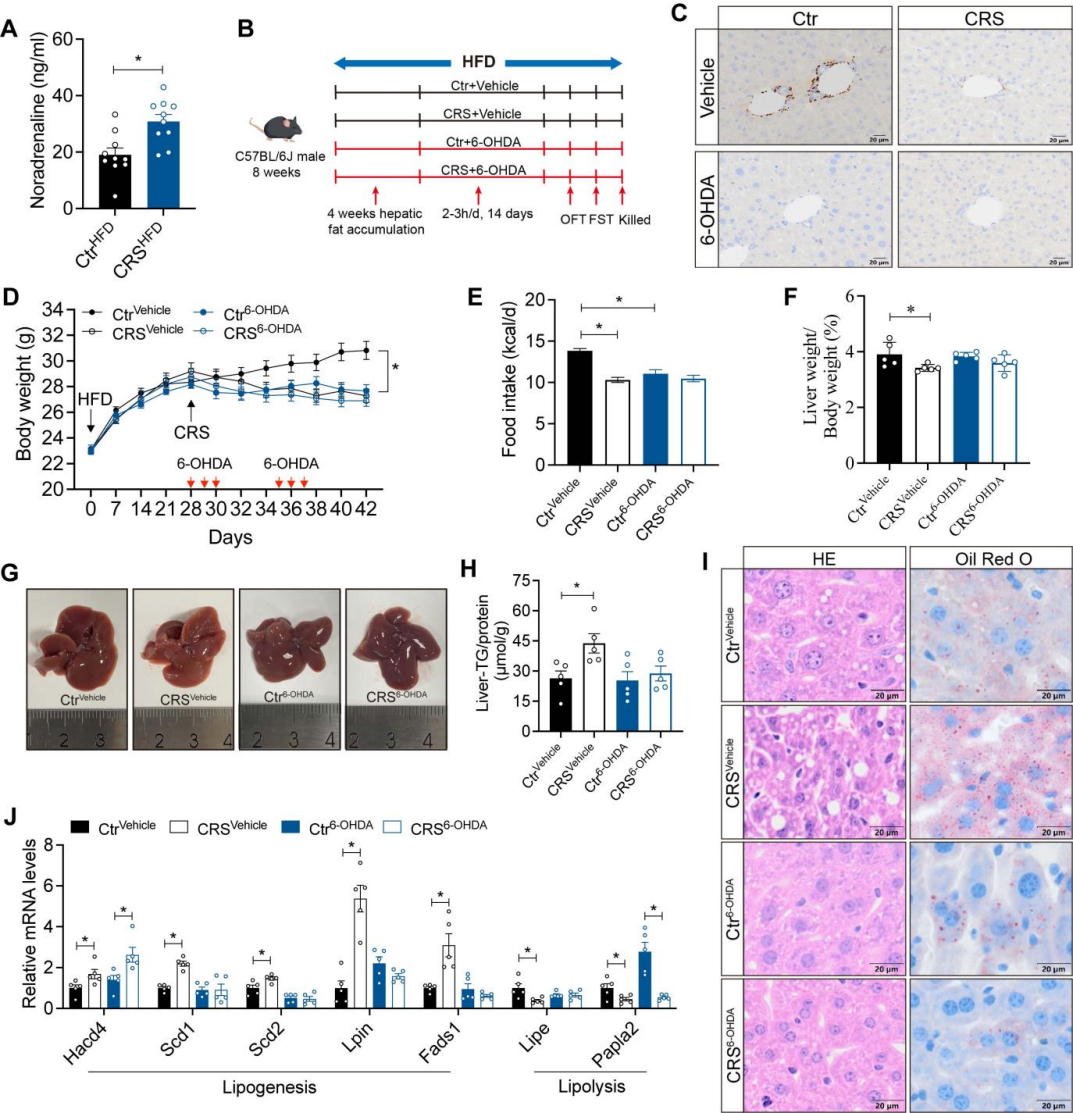
** Sympathetic hyperactivity mediates stress-induced hepatic steatosis.** (A) Serum NE levels, n = 10 per group. (B) Experimental scheme of 6-OHDA injection. (C) TH staining in liver section (scale bars, 20 μm). (D) Body weight, n = 10 per group. (E) Food intake, n = 10 per group. (F) Liver weight, n = 5 per group. (G) Liver anatomy. (H) Liver TG of saline and 6-OHDA injected mice, n = 5 per group. (I) H&E staining (scale bars, 20 μm) and Oil Red O staining (scale bars, 20 μm) of liver sections, n = 5 per group. (J) Relative mRNA levels in liver, n = 5 per group. The data are presented as mean ± SEM. *P < 0.05. Unpaired two-tailed Student's *t*-test and one-way ANOVA were used for statistical analysis.

Neuronal activity is essential for the stability and plasticity of nerve fiber^27^. Elevated serum NE levels in CRS mice (Figure 5A) are consistent with sympathetic activation^28^, ^29^. We hypothesized that chronic stress induces sustained sympathetic excitation, leading to compensatory degeneration of sympathetic structures. To further examine the contribution of hepatic sympathetic activity to hepatic lipid metabolism, we ablated sympathetic nerve using 6-OHDA (Figure 5B). TH staining in liver sections showed that 6-OHDA treatment markedly reduced hepatic sympathetic fibers, confirming effective sympathetic ablation (Figure 5C). Sympathetic blockade mice exhibited reductions in food intake and body weight similar to those observed in CRS mice (Figure 5D, E).

Hepatic sympathetic blockade modestly reduced hepatic lipid deposition and, importantly, abolished the effect of chronic stress on fatty liver formation (Figure 5F-I). Consistently, CRS-induced changes in hepatic metabolic gene expression were largely reversed after sympathetic blockade (Figure 5J). Notably, although sympathetic blockade ameliorated stress-related fatty liver, serum biochemical levels showed that sympathetic blockade impaired liver function, suggesting the complexity of the role of hepatic sympathetic nerves (Figure S1). Collectively, these data indicate that stress-induced sympathetic hyperactivity promotes hepatic lipid accumulation in the liver.

### Hepatic catecholamine resistance mediates stress-induced hepatic steatosis

**Figure 6:**
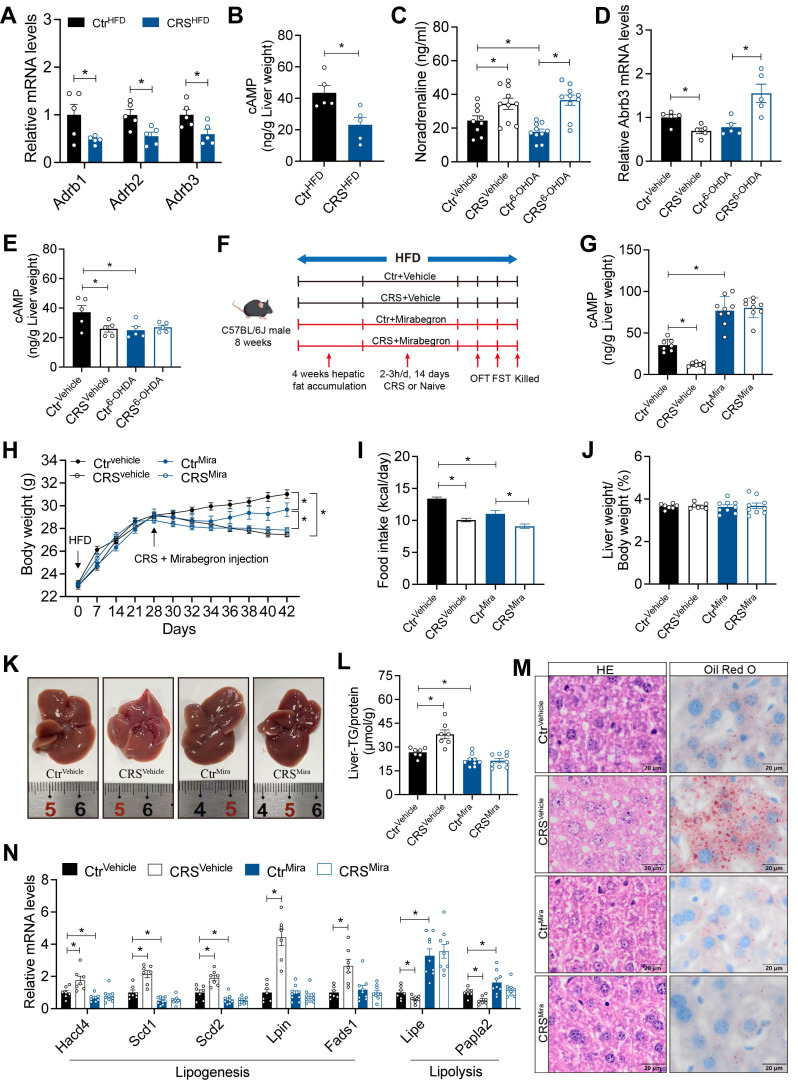
** Hepatic catecholamine resistance mediates stress-induced hepatic steatosis.** (A) Liver β-ARs mRNA levels of Ctr^HFD^ and CRS^HFD^ mice, n = 5 per group. (B) cAMP levels in liver extracts of Ctr^HFD^ and CRS^HFD^ mice, n = 5 per group. (C) Serum NE levels of 6-OHDA injection mice, n = 10 per group. (D) Liver β3-AR mRNA levels of 6-OHDA injection mice, n = 5 per group. (E) Hepatic cAMP levels of 6-OHDA injection mice, n = 5 per group. (F) Experimental scheme of mirabegron gavage, n = 7:7:9:9. (G) Hepatic cAMP levels of mirabegron gavage mice, n = 7:7:9:9. (H) Body weight of mirabegron gavage mice, n = 7:7:9:9. (I) Food intake of mirabegron gavage mice, n = 7:7:9:9. (J) Liver weight of mirabegron gavage mice, n = 7:7:9:9. (K) Liver anatomy of mirabegron gavage mice. (L) Liver TG of mirabegron gavage mice, n = 7:7:9:9. (M) H&E staining and Oil Red O staining of liver sections (scale bars, 20 μm), n = 7:7:9:9. (N) Liver relative mRNA levels of mirabegron gavage mice, n = 7:7:9:9. The data are presented as mean ± SEM. *P < 0.05. Unpaired two-tailed Student's *t*-test and one-way ANOVA were used for statistical analysis.

To further elucidate the mechanisms by which hepatic sympathetic activity mediates stress-related hepatic steatosis, we focus on the expression of hepatic AR. The mRNA levels of α-AR were increased (Figure S2A), whereas β-ARs were significantly decreased (Figure 6A). In adipose tissue, activation of β3-AR signaling is well recognized to promote lipolysis^16^. We therefore speculated that chronic stress induces sustained sympathetic hyperactivation and a compensatory reduction in hepatic β3-AR expression, consistent with catecholamine resistance. Further corroborating this, hepatic cAMP levels, a downstream readout of β3-AR signaling, were marked reduced in CRS mice (Figure 6B).

Notably, although sympathetic blockade effectively reduced NE release, chronic stress still increased serum NE levels, possibly due to increased NE release from the adrenal medulla induced by chronic stress (Figure 6C). Hepatic catecholamine resistance was alleviated by sympathetic blockade as revealed by the expression of β3-AR mRNA in the liver (Figure 6D). After 6-OHDA sympathectomy, chronic stress no longer adds suppression to β3-AR/cAMP signaling (Figure 6E). Stress-induced upregulation of Gi-coupled α2-AR signaling (Figure S2A) may further limit basal cAMP^30^, resulting in increased hepatic β3-AR mRNA (Figure 6D), but basal cAMP remained low (Figure 6E).

To investigated whether specific activation of β3-AR could alleviate the stress-induced hepatic steatosis, we used mirabegron to activate hepatic β3-AR signaling (Figure 6F). The increased hepatic cAMP levels in mirabegron-treated mice suggested effective β3-AR activation (Figure 6G). β3-AR activation reduced body weight and food intake (Figure 6H, I), without significant changes in serum ALT, AST, TG and TC levels (S2B-E). As expected, hepatic β3-AR activation alleviated stress-induced hepatic steatosis (Figure 6J-M) and reversed most of the stress-induced changes in mRNA expression of hepatic lipid metabolism genes (Figure 6N). Together, these results indicated that hepatic catecholamine resistance mediates stress-induced hepatic steatosis.

### Activation of PVH neurons mediates stress-induced hepatic lipid accumulation

**Figure 7:**
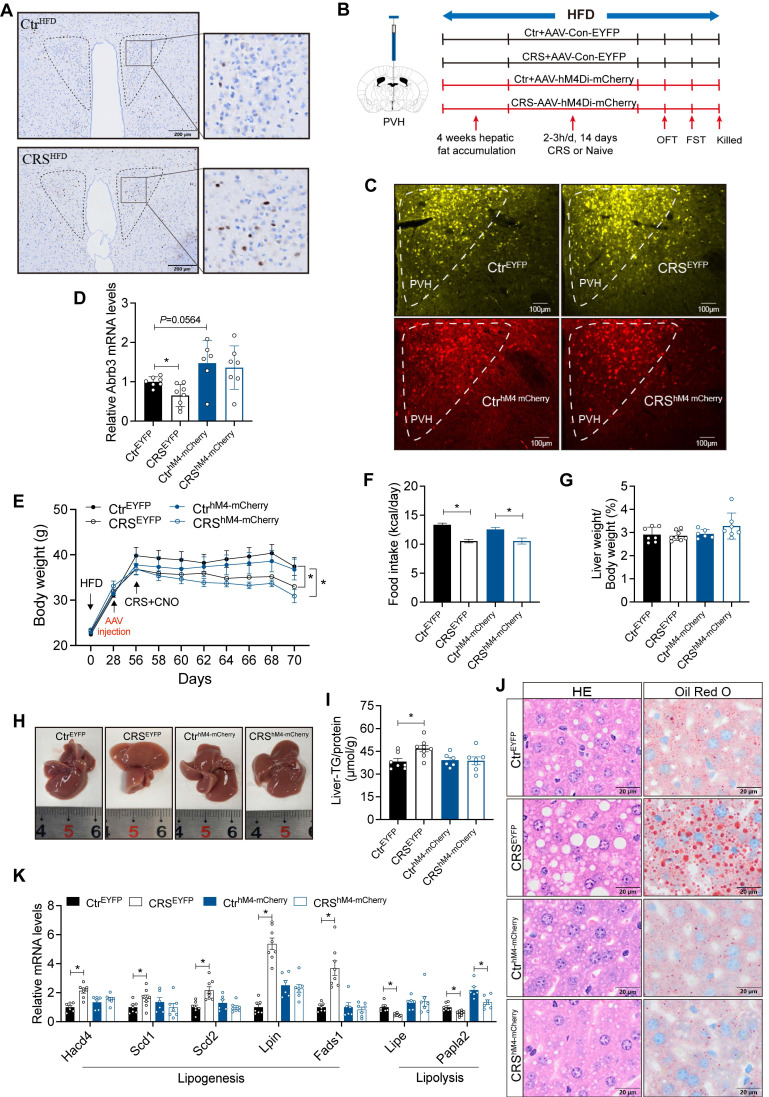
** Activation of PVH neurons mediates stress-induced hepatic lipid accumulation.** (A) c-Fos expression in PVH neurons, n=5. (B) Experimental scheme of PVH neurons inhibition. (C) Virus expression validation, (scale bars, 100 μm). (D) Liver β3-AR mRNA levels, n = 7:8:6:7. (E) Body weight, n=7:8:6:7. (F) Food intake, n=7:8:6:7. (G) Liver weight, n = 7:8:6:7. (H) Liver anatomy. (I) Liver TG, n = 7:8:6:7. (J) H&E staining (scale bars, 20 μm) and Oil Red O staining (scale bars, 20 μm) of liver sections, n = 7:8:6:7. (K) Liver relative mRNA levels, n = 7:8:6:7. The data are presented as mean ± SEM. *P < 0.05. Unpaired two-tailed Student's *t*-test and one-way ANOVA were used for statistical analysis.

PVH regulates sympathetic outflow from the brain to peripheral tissues^31^. To further support this, we injected PRV into the liver (Figure S3A). GFP was detected in multiple brain regions including PVH, confirming that PVH had a projection effect on hepatic sympathetic nerves (Figure S3B-I). Moreover, the activity of PVH neurons was increased in CRS mice (Figure 7A). These findings suggest that PVH neurons may contribute to stress-related hepatic steatosis.

To investigate whether the activity of PVH neurons is required for stress-induced fatty liver, we employed chemogenetic techniques to inhibit PVH neurons (Figure 7B, C). PVH neuronal inhibition modestly enhanced hepatic β3-AR signaling and reversed CRS-associated catecholamine resistance (Figure 7D). PVH neuronal inhibited mice and control mice exhibit similar trends in body weight and food intake in response to CRS (Figure 7E, F), with no significant changes in serum ALT, AST, TG and TC levels (Figure S4). As expected, inhibition of PVH neurons ameliorated CRS-induced hepatic lipid accumulation (Figure 7G-J) accompanied by reversal of abnormal expression of lipid metabolism genes in liver (Figure 7K).

### CeM-PVH projections mediate chronic stress-induced hepatic lipid dysregulation

**Figure 8:**
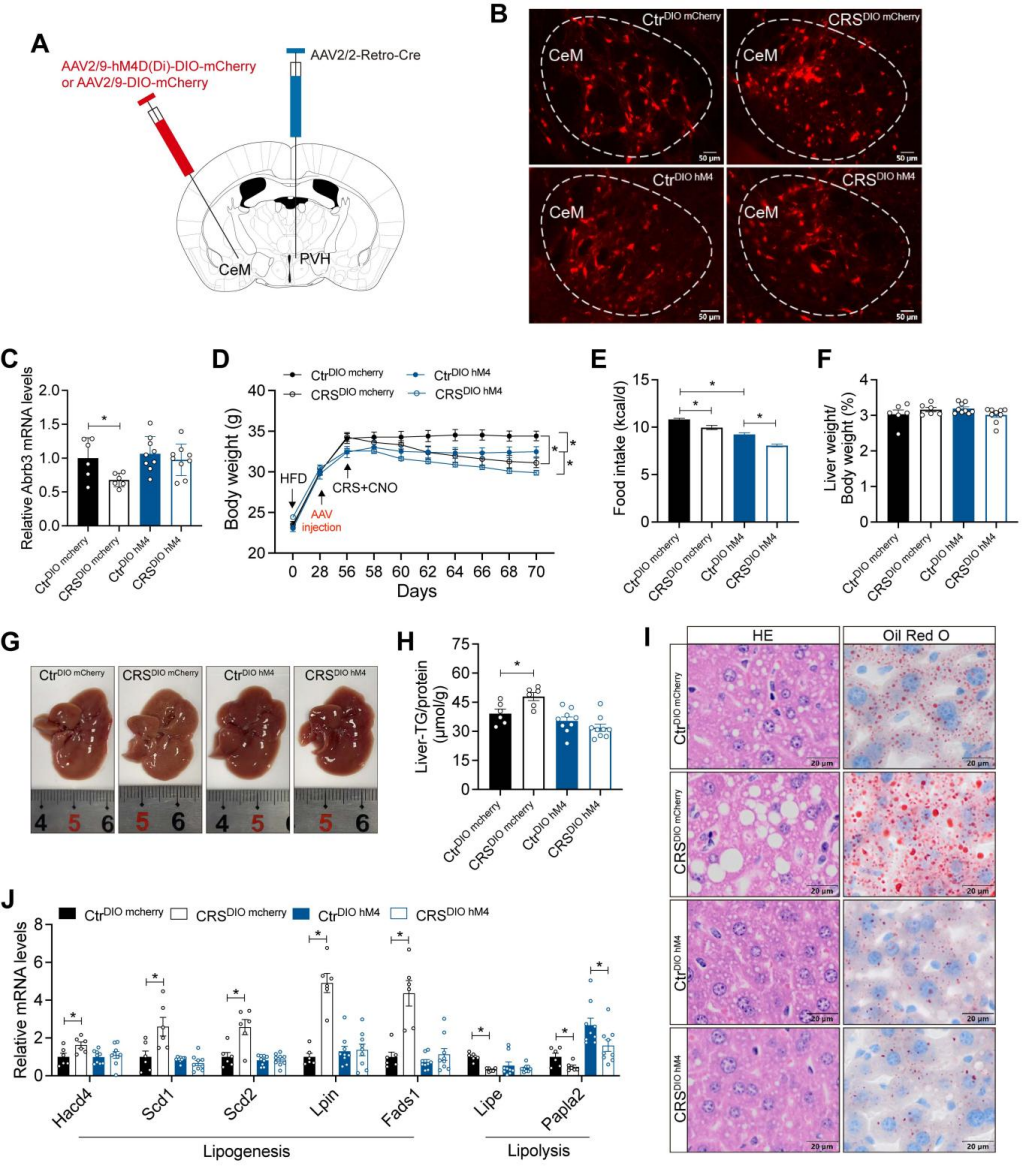
** CeM-PVH projection mediates chronic stress-induced hepatic lipid dysregulation.** (A) Experimental scheme of CeM-PVH projection inhibition. (B) Virus expression validation (scale bars, 50 μm). (C) Liver β3-AR mRNA levels, n = 6:6:9:9. (D) Body weight, n=6:6:9:9. (E) Food intake, n=6:6:9:9. (F) Liver weight, n = 6:6:9:9. (G) Liver anatomy. (H) Liver TG, n = 6:6:9:9. (I) H&E staining (scale bars, 20 μm) and Oil Red O staining (scale bars, 20 μm) of liver sections, n = 6:6:9:9. (J) Relative mRNA levels in liver, n = 6:6:9:9. The data are presented as mean ± SEM. *P < 0.05. Unpaired two-tailed Student's *t*-test and one-way ANOVA were used for statistical analysis.

Retrograde liver tracing revealed robust labeling in the CeM, a brain region closely implicated in stress processing (Figure S3F, G). We therefore hypothesized that CeM projections to the PVH contribute to stress-induced hepatic steatosis. To test whether CeM-PVH projection is required for stress-induced impairment of hepatic lipid homeostasis, we inhibited CeM neuron that project to PVH (Figure 8A). mCherry fluorescence in CeM suggested that our previous hypothesis of projection from CeM to PVH is reasonable (Figure 8B). Inhibition of CeM-PVH projection abolished catecholamine resistance caused by CRS (Figure 8C). Moreover, this manipulation did not alter CRS-induced reductions in body weight and food intake (Figure 8D, E), nor did it alter ALT, AST, TG, and TC levels in serum (Figure S5). As expected, inhibited CeM-PVH projection reversed the effects of chronic stress on hepatic lipid accumulation (Figure 8F-I) accompanied by reversal of abnormal expression of lipid metabolism genes (Figure 8J). Together, these results indicate that CeM projecting to PVH neurons are required for chronic stress to impair hepatic lipid homeostasis.

### The activity of CRH neurons in PVH mediates stress-induced hepatic lipid dysregulation

**Figure 9:**
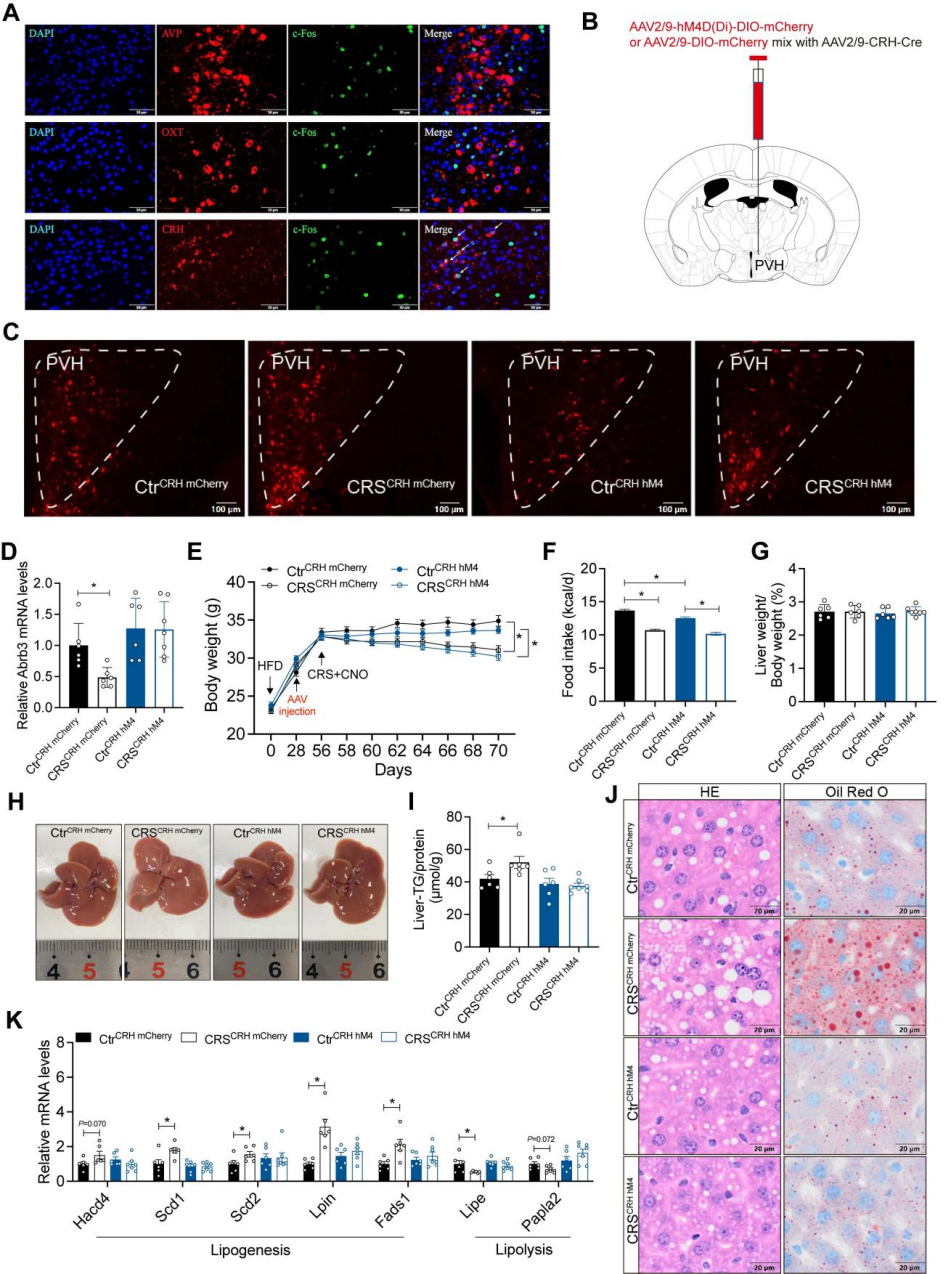
** The activity of CRH neurons in PVH mediates chronic stress-induced hepatic lipid dysregulation.** (A) Immunofluorescence co-localization. n = 5 per group. (B) Experimental scheme of inhibiting CeM-CRH^PVH^ projection. (C) Virus expression validation (scale bars, 50 μm). (D) Liver β3-AR mRNA levels, n = 6:6:6:7. (E) Body weight, n=6:6:6:7. (F) Food intake, n = 6:6:6:7. (G) Liver weight, n = 6:6:6:7. (H) Liver anatomy. (I) Liver TG, n = 6:6:6:7. (J) H&E staining (scale bars, 20 μm) and Oil Red O staining (scale bars, 20 μm) of liver sections, n = 6:6:6:7. (K) Relative mRNA levels in liver, n = 6:6:6:7. The data are presented as mean ± SEM. *P < 0.05. Unpaired two-tailed Student's *t*-test and one-way ANOVA were used for statistical analysis.

**Figure 10:**
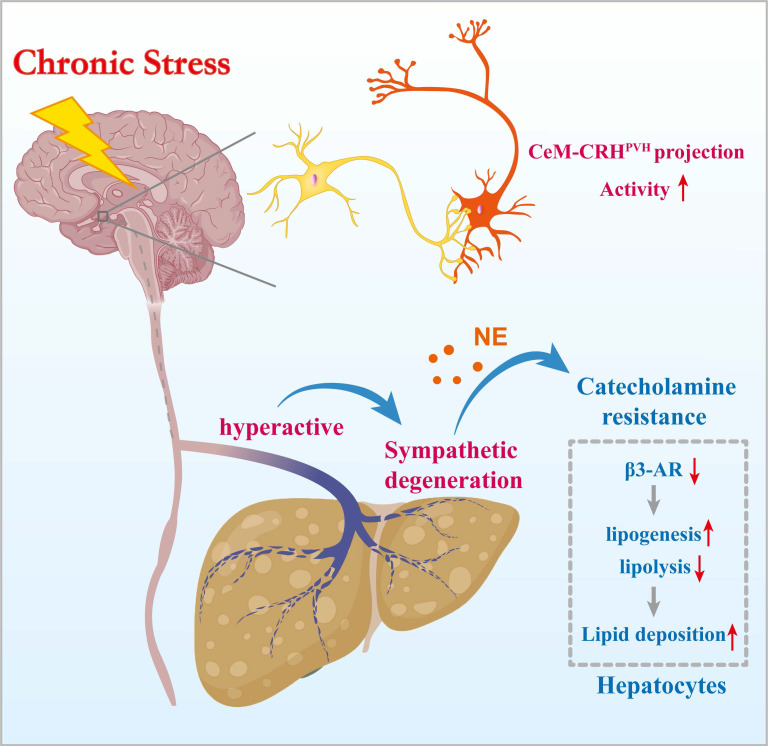
** Chronic stress induces hepatic steatosis via brain-hepatic sympathetic axis mediated catecholamine resistance**.

To identify the specific PVH neuronal types involved in stress-related hepatic lipid accumulation, we mapped activated neurons in the PVH of CRS mice. The results showed that chronic stress mainly activated CRH^PVH^ neurons, rather than AVP and OXT neurons (Figure 9A). We next used chemogenetic techniques to selectively inhibit CRH^PVH^ neurons (Figure 9B, C). This manipulation abolished catecholamine resistance caused by CRS (Figure 9D). Furthermore, inhibited CRH^PVH^ neuronal activity did not alter CRS-induced reductions in body weight and food intake (Figure 9E, F), nor did it alter ALT, AST, TG, and TC levels in serum (Figure S6). As expected, CRS-induced hepatic lipid dysregulation was reversed by inhibiting CRH^PVH^ neuronal activity (Figure 9G-J), accompanied by reversal of abnormal expression of lipid metabolism genes (Figure 9K), suggested that CRH^PVH^ neuronal activity is required for chronic stress to impair hepatic lipid homeostasis. Collectively, our findings uncover a CeM-CRH^PVH^-hepatic sympathetic-catecholamine axis in stress-induced hepatic lipid dysregulation (Figure 10).

## Discussion

Epidemiological studies have long associated chronic stress with adverse liver outcomes, yet mechanistic evidence has been limited^3^, ^32^-^34^. Here, we show that chronic stress induces hepatic steatosis through a CeM-CRH^PVH^-hepatic sympathetic-catecholamine axis. These findings connect epidemiological observations to mechanistic insight. We identify hepatic catecholamine resistance as a mediator that translates sustained central stress signaling into impaired lipid mobilization and pathological lipid accumulation.

A prevailing mechanistic hypothesis proposes that chronic stress enhances sympathetic tone, thereby stimulating adipose tissue lipolysis^35^. In line with this framework, we observed that CRS perturbs systemic lipid metabolic cues, including elevated plasma FFA and coordinated alterations in adipokines (Figure 3A-D). These endocrine signatures are consistent with stress-associated metabolic dysregulation and could, in principle, facilitate hepatic lipid accumulation. However, our adipose sympathetic denervation experiments demonstrate that adipose sympathetic lipolysis is not the dominant driver of stress-related hepatic lipid accumulation (Figure 3). This dissociation between peripheral lipid mobilization and hepatic lipid deposition suggests that chronic stress establishes a liver-permissive (or even liver-autonomous) steatogenic state that cannot be explained by substrate oversupply alone. Thus, the key conceptual advance is that stress-induced steatosis reflects an active hepatic metabolic program rather than a passive consequence of adipose-derived FFA overflow.

Catecholamine resistance is well described in adipose tissue under conditions such as obesity, insulin resistance, and chronic inflammation, where it is characterized by reduced β-AR expression, impaired cAMP signaling, and diminished lipolysis^36^. Our findings extend this concept to the liver, showing that sustained PVH-driven sympathetic activation during chronic stress leads to β-AR desensitization. This hepatic catecholamine resistance limits lipolysis and promotes lipogenesis, tipping the balance toward lipid retention. Moreover, the observed structural degeneration of hepatic sympathetic fibers may further exacerbate functional desensitization, forming a feed-forward loop of impaired catecholamine signaling and progressive steatosis. Our study provides the first direct evidence of stress-induced sympathetic degeneration in the liver.

Mechanistically, the paradoxical shift from “fat-burning” to “fat-storing” outcomes may involve several processes: (i) sustained norepinephrine exposure causing β-AR internalization and degradation; (ii) chronic excitatory drive inducing metabolic stress and sympathetic terminal injury; and (iii) prolonged sympathetic activation triggering local inflammation and oxidative stress. These adaptive responses may initially protect against lipotoxicity but ultimately predispose to hepatic steatosis. We also observed reciprocal upregulation of hepatic α-ARs (Figure S2A). This receptor subtype-specific remodeling suggests a functional shift in adrenergic signaling within the liver, with important hemodynamic consequences. α-AR activation-particularly via α1-subtypes-is well known to induce vasoconstriction by acting on vascular smooth muscle cells, thereby increasing vascular resistance^37^. Such an effect provides a plausible explanation for the marked vascular constriction visualized in our tissue clearing experiments (Figure 4A). This imbalance could reduce sinusoidal perfusion and oxygen delivery, creating localized hypoxic microenvironments that impair lipid homeostasis in hepatocytes^38^.

Clinically, our findings highlight psychosocial stress as a modifiable risk factor for MASLD progression. They also suggest therapeutic strategies: restoring β-adrenergic sensitivity (e.g., via β3-AR agonists such as mirabegron), or modulating CRH^PVH^ neuronal activity. Hepatic β-adrenergic responsiveness may also serve as a biomarker for patient stratification. Beyond pharmacological strategies, neuromodulation and behavioral stress reduction represent complementary approaches.

**Limitations** should be acknowledged. The molecular basis of hepatic catecholamine resistance warrants further investigation, including receptor profiling, downstream signaling analysis. Translation to human MASLD will require *in vivo* assessments of hepatic sympathetic activity and catecholamine responsiveness in patients. Furthermore, sex differences, genetic background, and variations in stress paradigms may influence the magnitude and nature of this response, and these factors should be systematically explored.

Collectively, our study identifies a novel axis as a mechanistic bridge between chronic stress and hepatic steatosis. By extending the concept of catecholamine resistance from adipose tissue to the liver, and by revealing the structural vulnerability of sympathetic fibers under chronic overactivation. We provide a new framework for understanding how sustained central stress signaling remodels peripheral metabolism, and open new avenues for both mechanistic research and targeted interventions in stress-related liver disease.

## Supplementary Material

Supplementary figures.

## Abbreviations

6-OHDA: 6-hydroxydopamine hydrobromide
β3-AR: β3-adrenergic receptors
ALT: alanine Aminotransferase
ANOVA: one-way analysis of variance
AST: aspartate Aminotransferase
AVP: arginine vasopressin
CNO: clozapine-N-oxide
cAMP: cyclic adenosine monophosphate
CeM: medial central amygdaloid nucleus
CRH: corticotropin releasing hormone
CRS: chronic restraint stress
DAPI: 4',6-diamidino-2-phenylindole
DMSO: dimethyl sulfoxide
eWAT: epididymal white adipocyte tissue
ELISA: enzyme-linked immunosorbent assay
FFA: free fatty acid
Fads1: fatty Acid Desaturase 1
FST: force-swim test
GAPDH: glyceraldehyde-3-phosphate dehydrogenase
Hacd4: 3-droxyacyl-CoA dehydrogenase 4
HFD: high-fat diet
H&E: hematoxylin and eosin staining
sWAT: subcutaneous white adipocyte tissue
Lipe: lipase
Lpin1: phosphatidate phosphatase 1
MASLD: metabolic dysfunction-associated steatotic liver disease
ND: normal diet
NE: norepinephrine
OFT: open-field test
OXT: oxytocin
PBS: phosphate-buffered saline
Pnpla2: patatin-like phospholipase domain-containing protein 2
PRV: pseudorabies virus
PVH: paraventricular hypothalamus
Scd1: stearic acyl-CoA desaturase 1
Scd2: stearic acyl-CoA desaturase 2
TC: total cholesterol
TG: triglycerides
TH: hydroxylase
