# Supplementary material for: Chronic Stress Induces Hepatic Steatosis via Brain-Hepatic Sympathetic Axis Mediated Catecholamine Resistance
Source: Int J Biol Sci. 2026 Jan 8;22(3):1407–24. doi: 10.7150/ijbs.126058 (PMC12837740; doi:10.7150/ijbs.126058)
Supplement: Supplementary file 1 — Supplementary figures. [file ijbsv22p1407s1.pdf]

Supplemental Figures

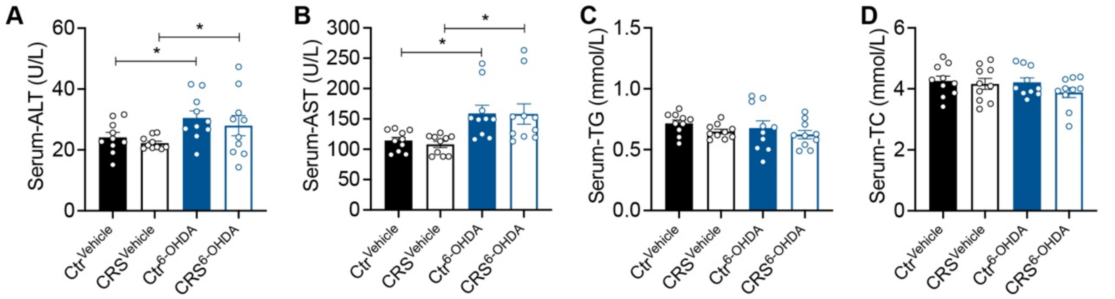

Figure S1. Serum biochemical indicators of sympathetic ablated mice

(A-D) Serum ALT, AST, TG and TC concentrations, n = 10 per group. The data are presented as mean ± SEM. *$P < 0.05$. Unpaired two-tailed Student's *t*-test was used for statistical analysis.

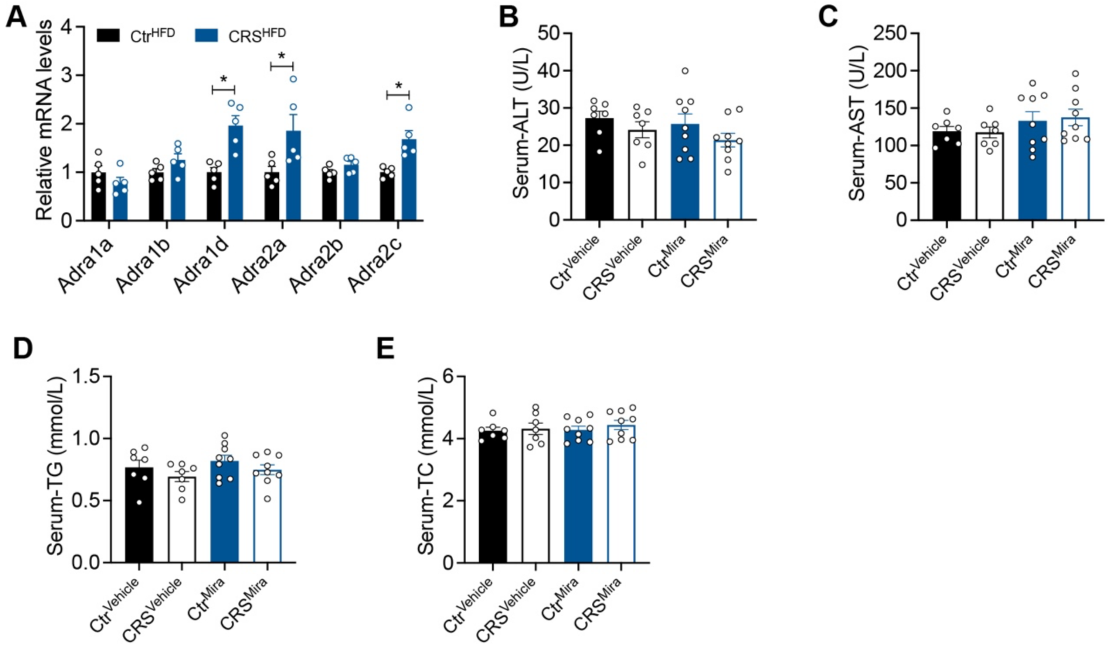

Figure S2. Hepatic α-ARs expression of CRS mice and serum biochemical indicators of mirabegron gavage mice

(A) Relative mRNA levels of α-ARs, n = 5 per group. (B-E) Serum ALT, AST, TG and TC concentrations, n = 7:7:9:9. The data are presented as mean ± SEM. *$P < 0.05$. Unpaired two-tailed Student's *t*-test was used for statistical analysis.

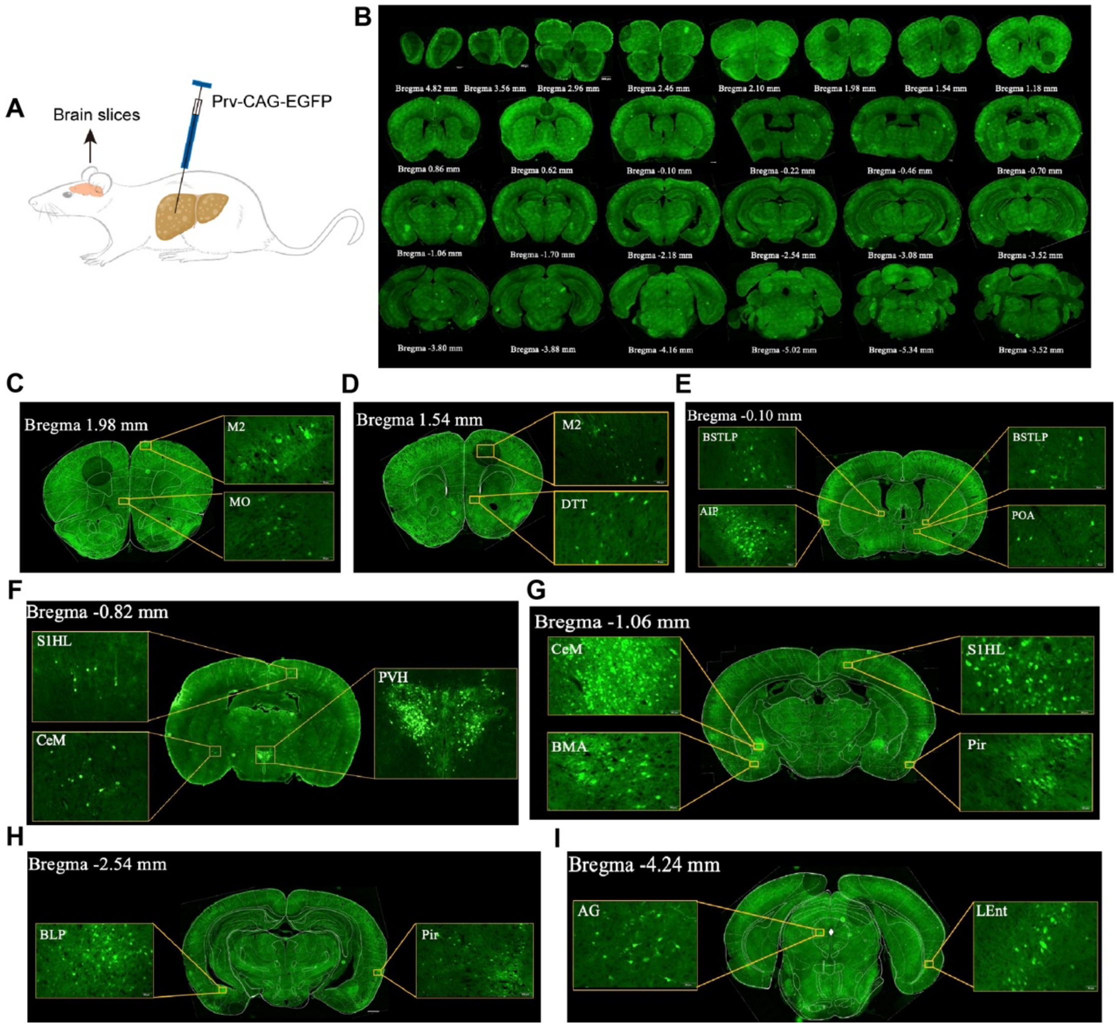

Figure S3. Retrograde tracing of hepatic neurons

(A) Experimental scheme of PRV injection. (B-I) GFP expression in brain regions.

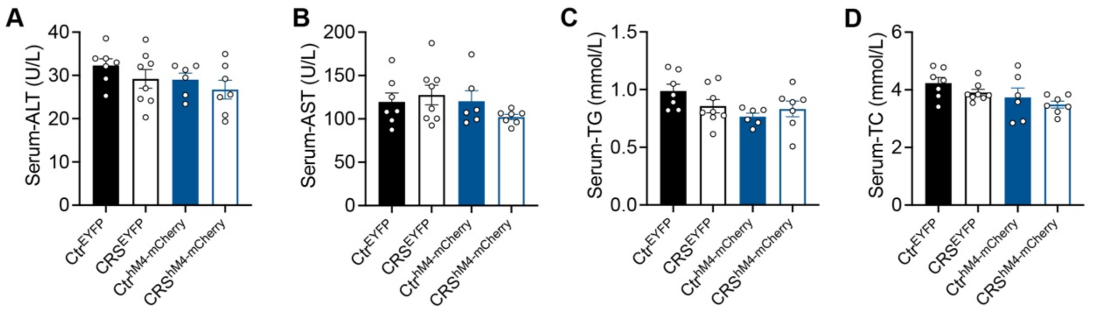

Figure S4. Serum biochemical indicators of PVH neuronal activity inhibited mice

(A-D) Serum ALT, AST, TG and TC concentrations, n=7:8:6:7. The data are presented as mean ± SEM. *$P < 0.05$. Unpaired two-tailed Student's *t*-test was used for statistical analysis.

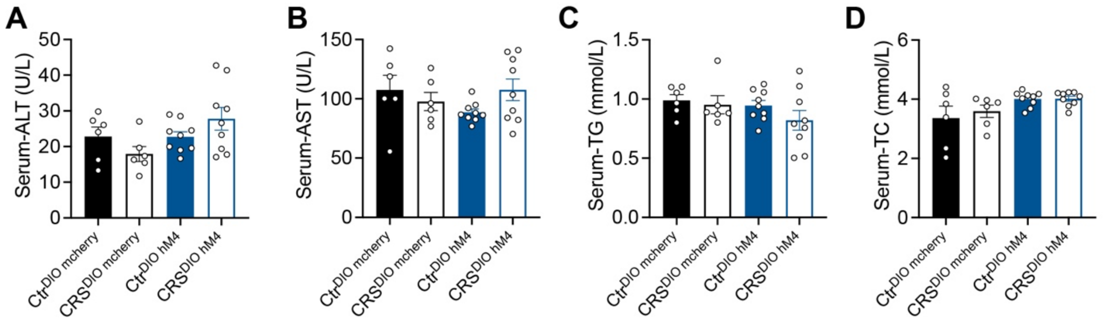

Figure S5. Serum biochemical indicators of CeM-PVH projection inhibited mice

(A-D) Serum ALT, AST, TG and TC concentrations, n=6:6:9:9. The data are presented as mean ± SEM. *$P < 0.05$. Unpaired two-tailed Student's *t*-test was used for statistical analysis.

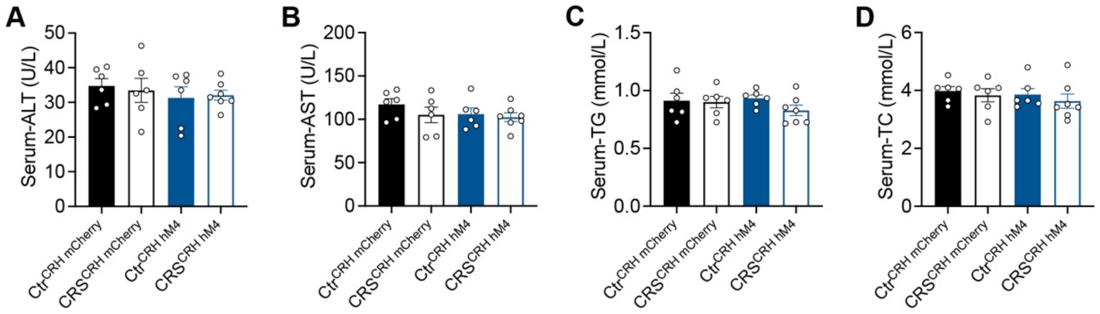

Figure S6. Serum biochemical indicators of CRH^{PVH} neuronal activity inhibited mice

(A-D) Serum ALT, AST, TG and TC concentrations, n=6:6:6:7. The data are presented as mean ± SEM. *P < 0.05. Unpaired two-tailed Student's *t*-test was used for statistical analysis.
